# Steroid-induced ischemic bone necrosis of femoral head: Treatment strategies

**DOI:** 10.12669/pjms.312.6592

**Published:** 2015

**Authors:** Bin Wu, Zhong Dong, Shuyuan Li, Hongmei Song

**Affiliations:** 1 Bin Wu, FCPS. Second People’s Hospital, Fujian University of Traditional Chinese Medicine, Fuzhou 350003, Fujian Province, China; 2 Zhong Dong, MD. Second People’s Hospital, Fujian University of Traditional Chinese Medicine, Fuzhou 350003, Fujian Province, China; 3 Shuyuan Li, PhD. Second People’s Hospital, Fujian University of Traditional Chinese Medicine, Fuzhou 350003, Fujian Province, China; 4 Hongmei Song, FCPS. Second People’s Hospital, Fujian University of Traditional Chinese Medicine, Fuzhou 350003, Fujian Province, China

**Keywords:** Steroid, Ischemic bone necrosis, Vasculature, Bone collapse, Arthoplasty

## Abstract

Fat hypertrophy, intravascular coagulation, and fat emboli are important risk factors of steroid-induced ischemic bone necrosis (SI-IBN) which may develop during the initial one year after commencing the use of steroids. This pathology is best studied by MRI, particularly for its staging. The cautious strategies such as low dose, oral route, short duration of steroid usage, use of steroid sparing agent, and alcohol avoidance should be followed as a traditional therapy. The objective of this review article was to recognize and evaluate various Treatment strategies for steroid-induced ischemic bone necrosis of femoral head.

**Literature Search:**

Various electronic databases including PubMed, Google and Cochrane library were comprehensively searched for articles on steroid-induced ischemic bone necrosis of femoral head and its treatment strategies. Ninety four articles were reviewed, examined and importantly appraised and the most appropriate 32 papers were used to write this review article.

**Conclusion:**

Bisphosphonates, alendronate, and hyperbaric oxygen (HBO) treatments have been reported to be effective against IBN. To recommend the regular use of bisphosphonate in IBN patients, more evidences with a larger number of patients are required to verify its therapeutic effectiveness. Core decompression, osteotomy, bone graft and tantalum rod are the surgical approaches for the management of IBN. Advance form of IBN (bone tissue collapse) is advised to be treated with arthroplasty which should be durable, particularly in young patients.

## INTRODUCTION

**Fig.1:**
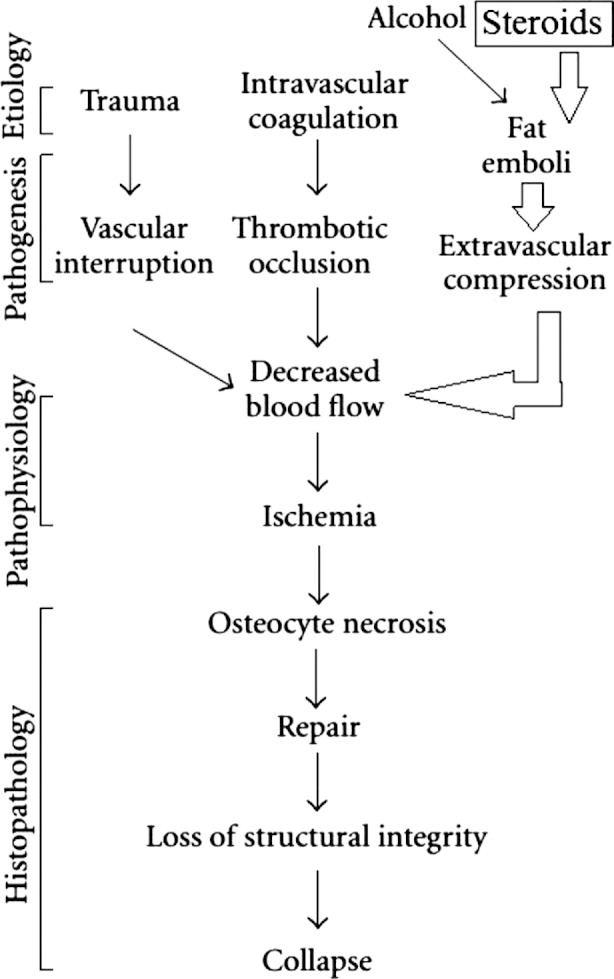
Graphical illustration of the development of steroid-induced ischemic bone necrosis of femoral head.

Ischemic bone necrosis (IBN), or osteonecrosis, is the *in situ*, progressive death of a bone fragment, possibly due to obstruction in blood supply and destruction of various cells such as fat cells and osteocytes. This condition may hinder the patient’s mobility due to pain that is aggravated by physical activity. With passage of time, patients may suffer from joint pains at rest also. Thus, early diagnosis of this disease is crucial due to limited treatment options for advanced disease state. On the basis of some underlying reason of necrosis, there are two types of IBN, i.e. primary and secondary IBN. The reason for IBN, sometimes, can be identified (secondary IBN), while primary or idiopathic IBN prevails when no comprehensive cause can be found.^1^-^3^ Trauma (e.g. fracture of the femoral head) is the most common etiology of secondary IBN. Other reasons for secondary IBN include hemoglobinopathies, dysbaric osteonecrosis, local infiltrative disease, hypercortisolism, alcohol consumption, pancreatitis, chronic renal failure, cigarette smoking, collagen vascular diseases, and congenital and developmental (Fig.1).^2^-^12^

Steroid-induced IBN is not mechanistically understood. It is however, hypothesized that obstruction in blood supply and destruction of various cells such as fat cells and osteocytes produces IBN. The obstruction in blood supply is produced by intravascular coagulation and fat emboli. Fat cell hypertrophy (FCH) and osteocyte apoptosis destroy the fat cells and osteocytes. These changes results in the compromised vasculature of bone and bone marrow. This pathology leads to IBN resulting in the failure of mechanical strength of bone.^6^

Pain is a characteristic indication in a patient with IBN. In the start of physical activity, pain intensity is generally less, but it aggregates with continued movements. The first line diagnostic test for IBN is conventional radiography that is comparatively convenient and economical. In later stage of IBN, the presence of ‘crescent sign’ reveals the occurrence of subchondral collapse as an abnormal finding in IBN. Other abnormal finding in IBN includes sclerotic modification and abnormal contours in femoral head. As a limitation of this approach, early IBN lesions are non-detectable through this radiological modality and for diagnosis of early IBN lesions, bone scintigraphy is a useful approach.^11^ Bones with early IBN are manifested with augmented osteoblastic activity and blood flow. This change is easily detectable by bone scintigraphy. The radioisotope uptake is reduced in the necrotic region of the bone in later stage of IBN but the situation is reversed in the surrounding subchondral bone in the proximity of IBN. Scintigraphy is also useful for the detection of abnormalities when pain at multiple bone and joint sites is complained. The limitations of this modality are poor spatial resolution, high radiation dose, less specificity for IBN, and impracticability for prognostic lesions.^13^

For the detection of IBN, magnetic resonance imaging (MRI) is considered the most sensitive approach. In T1 image, single line is the early detectable change that differentiates between normal and osteonecrotic bone. In T1 image, there may be two density lines representing the augmented vascularity of granulation tissue.^14^ The quantification of the affected area for the determination of the extent of IBN is the advantage of MRI over other modalities.^15^ High cost is the limitation of MRI technique, particularly if multiple sites are scanned.

**Fig.2:**
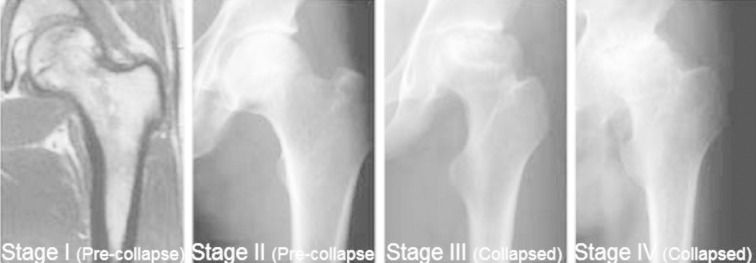
Ficat classification of ischemic bone necrosis of femoral head.

For proper prognosis and effective therapy, it is crucial to conclude accurate classification of IBN. In this context, various classification systems for femoral head IBN have been reported. One of the systems is Ficat 4-stage classification of IBN on the basis of radiographic observations (Fig.2). In comparison to normal radiograph (stage I), stage II is characterized by the indications of bone remodeling e.g. cystic and osteosclerotic sites with no effect on femoral head contour. The flattened femoral head or subchondral collapse is classified as stage III. The stage IV describes secondary degenerative transformation in acetabulum and the narrowed joint space.^16^

Second system considers the quantification of femoral head describing 6-stage classification of IBN in both, early and late stages on the basis of radiography, bone scintigraphy or MRI observations.^17^ Moreover, Association Research Circulation Osseous (ARCO) designed the latest, 4-stage classification system.^18^ In stage 0, all histological findings are normal. In stage I, radiographic observations are normal but bone scintigraphy or MRI or both detect the early stage IBN. In stage II, radiographic observations indicate abnormalities (mottled femoral head and cyst formation) but none of the modality show collapse of bone tissue. The emergence of ‘crescent sign’ is an indicator of stage III in which all diagnostic modalities reveal separation between subchrondral plate and the necrotic bone. The stage IV describes collapse of bone tissue and emergence of secondary degenerative transformation in acetabulum.^19^ The literature survey showed various studies conducted on systemic lupus erythematosus (SLE) patients who were chronically treated with steroids.^20^-^29^

### Literature Search

Various electronic databases including PubMed, Google and Cochrane library were comprehensively searched for articles on steroid-induced ischemic bone necrosis of femoral head and its treatment strategies using the terms “femoral head”, “ischemic bone necrosis” and “steroid-induced ischemic bone necrosis of femoral head”. This literature search was made in English. Ninety four articles were reviewed, examined to avoid duplication and importantly appraised and the most appropriate 32 papers were used to write this review article.

## TREATMENT STRATEGIES

The treatment strategies of SI-IBN are affected by 3s, i.e. site, stage and size of IBN which help in deciding the nature of treatment efforts, i.e. surgical and non-surgical modalities.^30^

### Patient Counseling

Both the patient and the physician should minimize the risk factors of IBN. The cautious strategies such as low dose, oral route, short duration of steroid usage, use of steroid sparing agent, and alcohol avoidance should be followed. The patient should be instructed to reduce his weight and do bed rest. However, the conservative approach alone is inadequate to treat disease development. It is evident from a prospective study conducted on patients with hip IBN concluding that the conservative approach (20%) is less successful than surgical approach (70%). Therefore advance approaches should be followed as discussed below.^31^

### Use f drugs

Bisphosphonates, alendronate, hyperbaric oxygen (HBO), and coenzyme Q_10_ treatment have been reported to be effective against IBN.^10^,^18^,^32^-^34^ The mode of activity of bisphosphonates involves the enhanced osteoclasts apoptosis, the suppressed resorption of the osteoclasts, and diminished apoptosis of osteoblasts and osteocytes. For recommending the regular use of bisphosphonate in IBN patients, more evidences with a larger number of patients are required to verify the therapeutic effectiveness of bisphosphonates. In addition, therapeutic use of alendronate in hip IBN has exerted an analgesic effect resulting in the improvement in patient’s mobility. Alendronate is also found to reduce the bone marrow edema.^33^ Another study showed some controversial evidence that alendronate used for a period of 25 weeks was found lesser effective (7%) in steroid-induced femoral head IBN than the placebo treatment (76%).^10^ Furthermore, HBO acts as analgesic by improving the oxygenation of hypoxic tissue, diminishing edema by augmenting the dissolved oxygen concentration, and producing vasoconstriction. Camporesi et al. reported considerable improvement in pain and mobility of IBN patient after using HBO intervention through healing of IBN.^34^ Moreover, coenzyme Q_10_ has been reported to possess antioxidant potential, that is why it is responsible for membrane stabilization and management of steroid-induced femoral head IBN.^35^ Another study has showed that pravastatin is effective to prevent steroid-induced osteonecrosis in rats. The possible mode action was the suppression of PPARγ expression and activation of Wnt signaling pathway.^36^ Additionally, Chen et al. documented that erythropoietin is useful against steroid-induced femoral head IBN. They attributed this activity to increased expression of vascular endothelial growth factor and suppression of apoptosis of osteocytes and osteoblasts by erythropoietin.^37^

### Surgical Approaches

Based on authentic radiographic assessment and disease stage, orthopedic surgical approach may be used to manage steroid-induced femoral head IBN at an earlier stage.^38^

### Core decompression

Core decompression deals with improving blood flow to bone making a drill hole in the femoral head to reduce intramedullary pressure. This approach is valuable in postponing the requirement for total hip arthroplasty. This approach is reported to be more useful for Ficat stage 1. In a study, the reported survival rate was 84%, 65%, and 47% for Ficat stage 1, stage 2 and stage 3 declaring that patients with earlier stage IBN had better progress. 32 It is also reported that core decompression method is better than conservative method in Ficat stage I, II and III hip.^16^ However, Koo et al. described core decompression to be useful for treating pain without affecting the time of bone tissue collapse in patients with IBN.^39^

### Osteotomy

Osteotomy involves the relocation of the necrotic site of bone from the weight bearing region of acetabulum for redistribution of weight to articular cartilage. It is worth mentioning that articular cartilage is sustained by healthy bone. Atsumi et al. proposed transtrochanteric anterior rotational osteotomy to have excellent results. The need of expertise is the limitation of this modality.^24^ It is also reported that transtrochanteric anterior rotational osteotomy has good results (76%) with Ficat stage II or III IBN. The lateral displacement of the greater trochanter is the disadvantage of this approach.^12^

### Bone graft

Subchondral bone or cartilage is given mechanical strength through bone grafting, alone or combining with other modalities such as osteotomy. Moreover, blood flow to the bone may also be improved through vascularized bone grafting which is more successful than cortical graft. The success of vascularized bone grafting could be assessed from two studies which reported that only 11% and 10.5% stage II hip IBN cases were required to undertake THR in a five year and ten year, respectively follow up period after vascularized fibular grafting.^6^ Post-operation weakness and pain are the limitations of this modality.^7^

### Tantalum rod

For the prevention of bone collapse in Steinberg stage I-III femoral IBN, the porous tantalum rod, manufactured with biocompatible material, is an excellent substitute of the necrotic bone.^31^ Its porosity is 75% which helps in swift bony in growth.^40^ Only 70% stage I-III hip IBN cases were required to undertake THR in a six year follow up period after insersion of tantalum rod.^9^ The tip of protrusion of the tantalum rod into the acetabulum may occur if the disease progresses. Other disadvantage of the tantalum rod is its difficult removal due to strong bonding with the surrounding bone.

### Joint replacement

Total hip replacement, hemiarthroplasty, and femoral resurfacing arthroplasty are advised in stage IV IBN which involves collapse of the femoral head. However, this modality faces high failure rate in patients with IBN compared with THR due to other causes like osteoarthritis.^32^ It could be due to the physical more active nature of young patients who are usual sufferers of IBN. Due to this limitation, the use of joint preserving methods should be investigated. The usage of highly cross-linked polyethylene and ceramic-on-ceramic are excellent examples of more wear resistant bearing surfaces as a joint preserving approach. According to a study, the annual penetration was <0.01 mm per year during seven year follow up in patients with THR using highly cross-linked polyethylene with a success rate of 71.68% hips with IBN.^24^

### Regenerative Medicine

For the improvement of treatment outcome, regenerative medicine is a new treatment modality which involves the use of cells, biomaterial scaffolds, and bioactive factors. For bone regeneration, there is great importance of biotechnological procedures of concentrated bone marrow aspirates, *ex vivo* expanded mesenchymal stem cells, and osteogenic or angiogenic growth factors (or both). To reduce the requirement of THR for the preservation of joint function, anterograde surgical approaches, including osteochondral transplantation, matrix-based autologous chondrocyte implantation, or the usage of acellular scaffolds alone has gained much importance.^41^ Stem cell-based regenerative treatments involve the use of adult tissue-derived, multipotent mesenchymal stem cells. Multipotent mesenchymal stem cells are believed to play a vital role in the maintenance of integrity of various tissues including bones. These cells possess capability of tissue regeneration after transplantation through mitotic multiplication and differentiation into multiple mesenchymal phenotypes, such as osteoblasts, chondrocytes, and adipocytes.^16^ On the other hand, growth factor-based strategies involve bone healing through many growth factors including insulin-like growth factor-1 and -2, transforming growth factor-β1, platelet-derived growth factor, and fibroblast growth factor-2 synthesized by osteogenic cells, platelets, and inflammatory cells. These growth factors reside in the bone matrix and are activated by various factors such as matrix metalloproteases and the acidic environment. The activated growth factors help in movement, multiplication, and differentiation of multipotent mesenchymal stem cells into osteoblasts or chondroblasts with an outcome of joint-preserving treatment of IBN.^10^ Table-I describes various stem cell- and growth factor-based regenerative treatments for IBN of the femoral head. Since the efficacy of core decompression is variable, bone marrow containing osteogenic precursors has been implanted into necrotic lesion of femoral head with IBN to test the influence of bone-marrow buffy coat grafting combined with core decompression. This joint therapeutic approach has proved very promising for the treatment of steroid-induced femoral head IBN in human patients with early stages of disease.^42^ Tian et al. reported that Toll-like receptor 4 signaling pathway of human immune system is involved in pathogenesis of steroid-induced femoral head IBN, therefore disruption of Toll-like receptor 4 signaling pathway was found effective to treat IBN.^43^

**Table I:** Stem cell- and growth factor-based regenerative therapies for avascular necrosis of the femoral head.

No.	Number of treated Cases	Unsuccessful cases (cases moved to THR)	Follow up time	Drawback of study	IBN stage	References
***Concentrated bone marrow aspirates***						
1	169	34	5-10 years	No control group	I-IV	1
2	10	1	2 years	Short term follow-up periods and low case numbers	I,II	4
3	13	3	5 years	Short term follow-up periods and low case numbers	I-III	9
***Application of ex vivo expanded autologous bone marrow derived stem cells***						
1	5	0	16 months	Short term follow-up periods and low case numbers, No control group	II	16
2	3	0	34 months	Short term follow-up periods and low case numbers	III,IV	27
3	4	0	2 years	Short term follow-up periods and low case numbers	II	28
***Osteogenic growth factors (Bone morphogenetic proteins)***						
1	15	3	53 months	No control group	I, II	10
2	21	4	4 years	Short term follow-up periods and low case numbers	I-III	18
3	39	15	3 years	No control group	III	10

## CONCLUSION

Surgery of the femoral head could be an appropriate therapy after its collapse. Core decompression is a better approach for the clinical management of early IBN of the femoral head as compared to non-operative and other operative treatment modalities. Regenerative medicineis aimed to preserve a physiological joint function. Advanced stages of IBN still need suitable surgical solution, especially for young patients who generally suffer from IBN and need THR. The limitations of both cell- and growth factor-based strategies include randomized clinical trials for the validation of their effectiveness in comparison with conventional treatment approaches.
